# Nuclear resonant inelastic X-ray scattering at high pressure and low temperature

**DOI:** 10.1107/S1600577515003586

**Published:** 2015-04-10

**Authors:** Wenli Bi, Jiyong Zhao, Jung-Fu Lin, Quanjie Jia, Michael Y. Hu, Changqing Jin, Richard Ferry, Wenge Yang, Viktor Struzhkin, E. Ercan Alp

**Affiliations:** a Department of Geology, University of Illinois at Urbana-Champaign, Urbana, IL 61801, USA; b Advanced Photon Source, Argonne National Laboratory, Argonne, IL 60439, USA; c Department of Geology Sciences, The University of Texas at Austin, Austin, TX 78712, USA; d Center for High Pressure Science and Technology Advanced Research (HPSTAR), Shanghai 201203, People’s Republic of China; e Institute of High Energy Physics, Chinese Academy of Sciences, Beijing 10090, People’s Republic of China; f Insitute of Physics, Chinese Academy of Sciences, Beijing 10090, People’s Republic of China; g HPSynC, Carnegie Institution of Washington, Argonne, IL 60439, USA; h Geophysical Laboratory, Carnegie Institution of Washington, Washington, DC 20015, USA

**Keywords:** nuclear resonant scattering, phonon density of states, high pressure, low temperature, iron-pnictides

## Abstract

A new synchrotron radiation experimental capability of coupling nuclear resonant inelastic X-ray scattering with the cryogenically cooled high-pressure diamond anvil cell technique is presented. The new technique permits measurements of phonon density of states at low temperature and high pressure simultaneously, and can be applied to studies of phonon contribution to pressure- and temperature-induced magnetic, superconducting and metal–insulator transitions in resonant isotope-bearing materials. In this report, a pnictide sample, EuFe_2_As_2_, is used as an example to demonstrate this new capability at beamline 3-ID of the Advanced Photon Source, Argonne National Laboratory. A detailed description of the technical development is given. The Fe-specific phonon density of states and magnetism from the Fe sublattice in Eu^57^Fe_2_As_2_ at high pressure and low temperature were derived by using this new capability.

## Abstract 2

The development of a novel experimental capability of nuclear resonant inelastic X-ray scattering at high pressure and low temperature is presented. The new capability is demonstrated by studying the Fe-specific phonon density of states and magnetism in Eu^57^Fe_2_As_2_.

## 1. Introduction   

Nuclear resonant inelastic X-ray scattering (NRIXS) was developed about two decades ago (Seto *et al.*, 1995 ▸; Sturhahn *et al.*, 1995 ▸). The technique uses Mössbauer isotopes as probes while using synchrotron radiation as the source of excitation. Elemental and isotopic selectivity, directional sensitivity and rich information content are hallmarks of this technique. The resulting partial phonon density of states are readily comparable with theoretical simulations *via* density functional theory (Lin *et al.*, 2011 ▸). Determinations of thermodynamic, vibrational and elastic properties such as sound velocity, vibrational entropy, kinetic energy and specific heat made the technique attractive to diverse fields such as physics, chemistry, biology, materials science and geology. The technique and its applications have been reviewed extensively by Sturhahn and Röhlsberger (Sturhahn, 2004 ▸; Röhlsberger, 2005 ▸). The technique has gained extensive momentum in recent years and the number of places to perform such measurements has proliferated. In the meantime, measurements of NRIXS spectra under extreme conditions of pressure (Mao *et al.*, 2001 ▸) and temperature (Chumakov & Sturhahn, 1999 ▸) have been performed. With the X-ray beam incident at grazing incidence angle, sample thickness has been reduced to a monolayer (Alp *et al.*, 2001 ▸; Stankov *et al.*, 2010 ▸). In an attempt to combine several environmental parameters, high pressure and high temperature have been achieved using laser heating in diamond anvil cells (DACs) (Zhao *et al.*, 2004 ▸; Lin *et al.*, 2005 ▸; Gao *et al.*, 2009 ▸; Jackson *et al.*, 2013 ▸). With a renewed interest in phonon physics due to the discovery of novel superconductivity in iron pnictides and chalcogenides (Boeri *et al.*, 2008 ▸; Hahn *et al.*, 2009 ▸; Sergueev *et al.*, 2013 ▸; Kobayashi *et al.*, 2013 ▸), it has become imperative to carry out NRIXS experiments under high-pressure (HP) and low-temperature (LT) conditions.

However, so far NRIXS has barely been measured simultaneously under HP and LT conditions due to some technical challenges. In a typical low-temperature and high-pressure experiment, a DAC is placed in a cryostat where cryogenic temperatures are achieved on the sample either by physical contact with a cold finger or by directly cooling through immersing the DAC in a cryogenic liquid. The vacuum shroud and thermal radiation shield on the cryostat greatly increase the distance from the sample to the silicon avalanche photodiode (APD) detectors, significantly reducing the counting rate and therefore increasing the data collection time. These technical difficulties make it unrealistic for NRIXS measurement at current synchrotron radiation facilities.

In this paper we describe a new NRIXS capability under HP–LT conditions at beamline 3-ID of the APS. Beamline 3-ID is a dedicated beamline for nuclear resonant scattering (NRS) studies (Alp *et al.*, 1994 ▸). NRS experiments are carried out during the standard operating mode with 24 electron bunches and 102 mA storage ring current. The bunch interval is 153 ns. At 3-ID, experimental capabilities for ^83^Kr, ^57^Fe, ^151^Eu, ^161^Dy and ^119^Sn resonant isotopes are available. The X-ray beam is monochromated by a high-heat-load monochromator and subsequently by a high-resolution monochromator (Toellner, 2000 ▸; Toellner *et al.*, 2006 ▸). The X-rays are then focused by Kirkpatrick–Baez mirrors to 15 µm diameter onto the sample in a DAC.

The NRS signal from the sample is collected using APD detectors (*e.g.* Kishimoto, 1992 ▸; Baron & Ruby, 1994 ▸; Baron, 2000 ▸) with an area of 1 cm × 1 cm with a typical time resolution of 1 ns. In synchrotron Mössbauer spectroscopy (SMS) experiments, one APD detector is placed to collect coherently scattered photons in the forward direction. In a typical HP NRIXS experiment, a panoramic DAC is used (Mao *et al.*, 2001 ▸). Wide openings in the DAC provide large solid angle for the nuclear fluorescence radiation of 14.4 keV and atomic fluorescence of Fe *K*_α_ and *K*_β_ radiation following an internal conversion (Gütlich *et al.*, 1978 ▸). The setup described here accommodates two APDs which are placed around the sample in a plane perpendicular to the incoming X-ray beam. An online X-ray diffraction system with a MAR3450 image plate detector is used to collect diffraction spectra from the sample or the pressure marker in order to determine the lattice parameters of the sample or to determine the pressure in the sample chamber (Gao *et al.*, 2009 ▸). An online ruby fluorescence system is available for *in situ* pressure measurements.

In the following sections we present details of the design and results of the NRIXS measurements at HPLT. By adapting the miniature panoramic DAC with a liquid helium flow cryostat for NRIXS experiment, we succeeded in measuring the phonon density of states at HP and simultaneously at LT with good counting rates. We can foresee that this new capability is readily applicable to other spectroscopic techniques such as X-ray Raman spectroscopy (Goncharov, 2012 ▸), resonant inelastic X-ray scattering (RIXS) (Rueff & Shukla, 2010 ▸), X-ray emission spectroscopy (Rueff *et al.*, 1999 ▸) and X-ray absorption spectroscopy (EXAFS and XANES) (Itiéf Baudelet *et al.*, 1992 ▸) in both transmission and fluorescence modes at high pressure and low temperature.

## 2. Instrumentation development   

**Figure 1:**
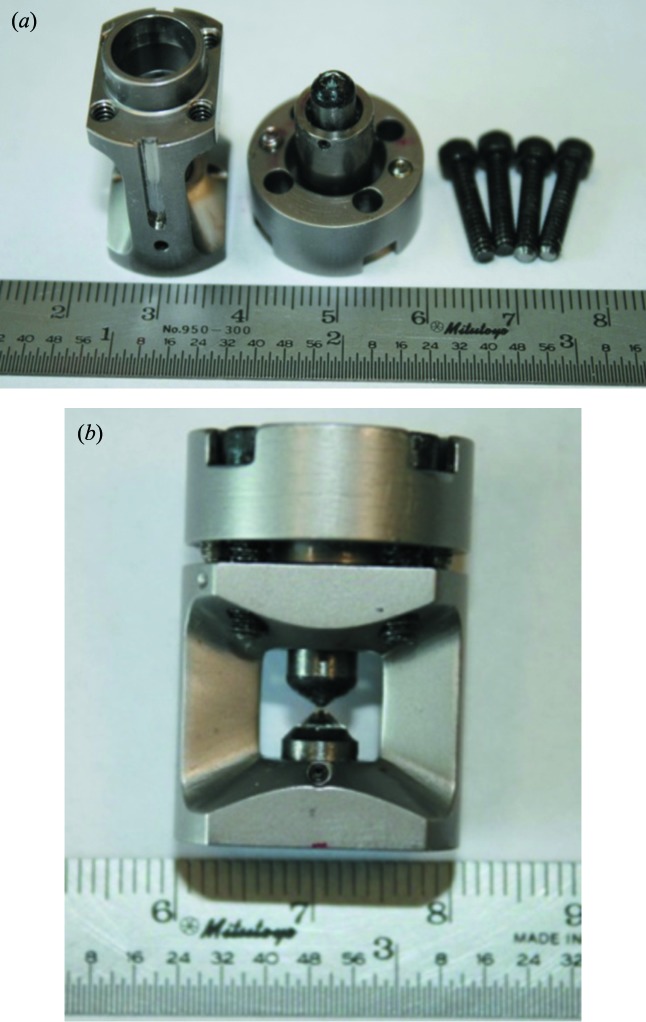
Images of the miniature panoramic DAC. (*a*) Photographs of the miniature DAC parts. (Left) Cylindrical piece. Two wide windows are cut to provide large solid angles for fluorescence signals reaching APD detectors. The dimension of the cylinder is reduced to 13.6 mm along the axis of the opening. A groove is made on the body to accommodate a silicon diode temperature sensor. (Middle) Piston of the DAC with a diameter of 20.3 mm. (Right) Four screws used on the piston for pressure application. (*b*) Photograph of an assembled DAC showing the wide windows.

In order to minimize the distance between the sample and the detector, we have developed a miniature panoramic DAC (Fig. 1 ▸). The DAC is made of hardened Vascomax 350 with a hardness of Rockwell C55, and was machined at the Mechanical Engineering Machine Shop of the University of Texas at Austin. The miniature DAC adopts a piston-cylinder type design shown in Fig. 1 ▸. Pressure is applied by tightening four screws on the piston side. The diameter of the DAC is 20.3 mm. Two openings are cut on the cylindrical part, each with 140° equatorial angle and 68° along the DAC axis for fluorescence signals reaching the APD detectors. A silicon diode sensor is placed in the groove of the cylindrical piece (Fig. 1*a* ▸) for measuring temperatures of the DAC body. As shown in Fig. 1(*a*) ▸, the cylindrical part is reduced to 13.6 mm along the openings so that when placed in a cryostat the distance to the APD located outside of the cryostat is reduced.

**Figure 2:**
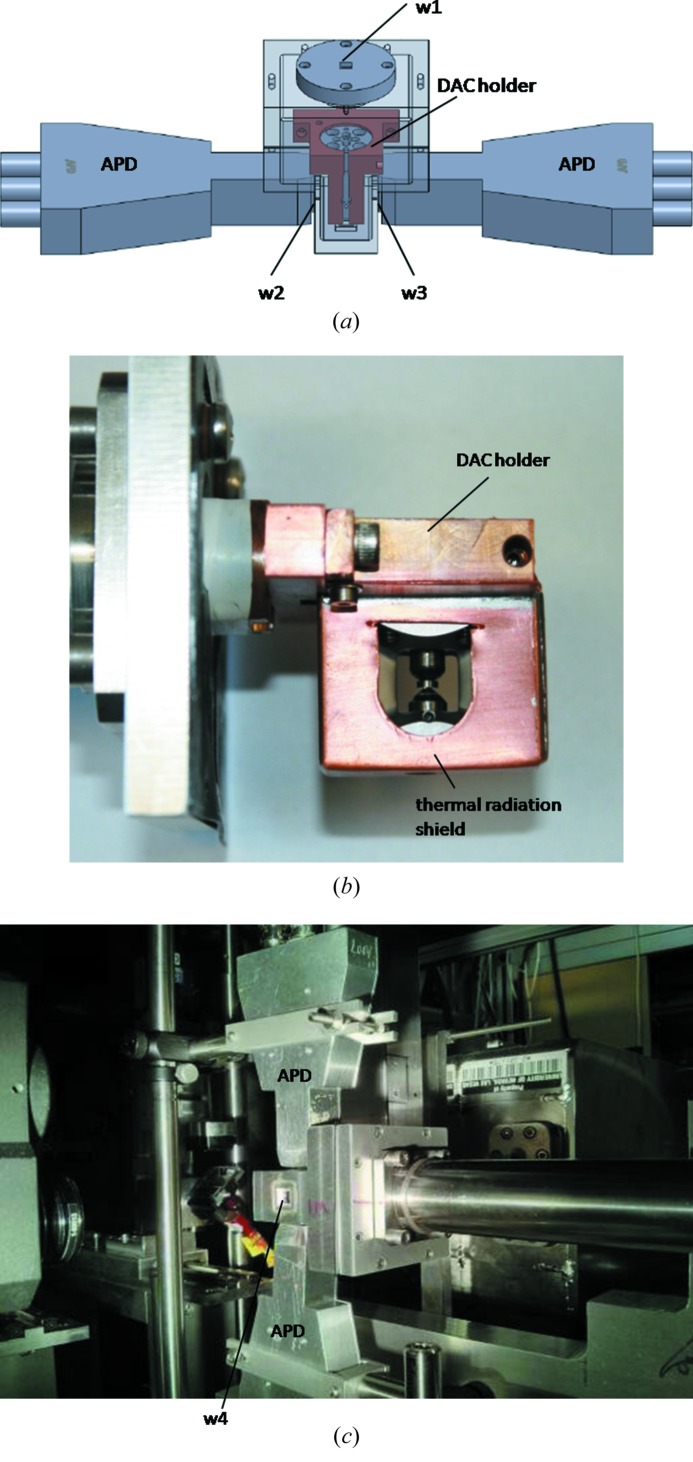
(*a*) Schematics of the mini-panoramic DAC inside the vacuum shroud of the cryostat with two APD detectors located outside the cryostat. The DAC is mounted on the cold finger of the cryostat by a copper DAC holder. The shape of the holder is designed to provide good thermal contact to the DAC. Three windows are visible in the schematics: w1, w2 and w3. Diamond window w1 has an area of 4 mm × 4 mm to allow incoming X-rays to reach the sample. Kapton windows w2 and w3 allow the inelastic scattering signals to reach the APDs. Each window has an area of 1 cm × 1 cm. The distance from the sample to the APD detectors is 12 mm. (*b*) Photograph of a thermal radiation shield and a DAC mounted on the cryostat. (*c*) Photograph of the HP–LT NRIXS setup at 3-ID-B. Two APDs are mounted on the top and bottom of the vacuum shroud of the cryostat. The window w4 shown provides optical access of the ruby fluorescence signals for pressure determination as well as allowing the measurement of SMS by a forward APD.

To achieve low temperature, a continuous-flow cryostat (Oxford, CF2102) is used. A DAC holder was made to accommodate the DAC inside the cryostat as shown in Fig. 2 ▸. Two APD detectors are placed outside of a vacuum shroud for the cryostat (Fig. 2 ▸). Four windows (w1, w2, w3, w4) are constructed on the vacuum shroud to allow X-ray beams to reach the sample, and allow the inelastic and forward signals to reach the APD detectors. A diamond window (w1) of area 4 mm × 4 mm and thickness 100 µm allows the incoming X-rays to reach the sample. Two windows (w2 and w3), each of area 1 cm × 1 cm, allow the inelastic scattering signals to reach the APD detectors. Kapton foil of thickness 2.5 µm is used as the window material for w2 and w3 to minimize the absorption. The size of these windows is compatible with the area of the APD detector. The NRIXS APDs cover the same opening angles from the DAC. The distance from the APD to the sample is about 12 mm. A sapphire window (w4) downstream of area 6 mm × 6 mm allows SMS measurements and also serves as an optical window for pressure determination using the ruby fluorescence technique at low temperature. The miniature DAC is mounted on the cold finger of the cryostat by a copper DAC holder (Fig. 2*a* ▸). This holder keeps the piston part of the DAC tight to achieve good thermal contact. A sleeve made of thin copper sheet covers the body of the DAC, which serves as a thermal radiation shield. Windows were cut on the sleeve to be compatible with those in the DAC allowing inelastic and forward signals to exit the cryostat. The temperature of the DAC is monitored by a Si diode thermometer placed in a groove of the DAC (see Fig. 1*a* ▸). Apiezon N grease is applied to the sensor to ensure good thermal contact between the sensing surface of the Si diode and the DAC. The temperature sensor is held in place with a Teflon screw threaded on the DAC holder shown in Fig. 2(*a*) ▸. When changing pressure, the sample is warmed up to room temperature by stopping the liquid helium flow and applying a heater on the cryostat. After venting the cryostat, the circular flange on top of the shroud (w1) in Fig. 2(*a*) ▸ is then removed to give access to the four screws on the piston side of the DAC without the necessity of removing the whole vacuum shroud from the cryostat.

## 3. Experiments   

To demonstrate the new capability of NRIXS at HP–LT, we have performed NRIXS as well as SMS experiments under HP–LT conditions on a Fe-pnictide sample EuFe_2_As_2_ to investigate the Fe-specific phonon density of states and the local magnetism of the Fe sublattice. In recent years, Fe-based superconductors (FeSCs, including Fe-pnictides and Fe-chalcogenides) with superconducting temperature (*T*_C_) as high as 65 K have rekindled research interest in high-temperature superconductivity (for a review, see Paglione & Greene, 2010 ▸). The parent compounds of these superconductors exhibit a tetragonal to orthorhombic structural transition accompanied by a magnetic transition forming spin density wave (SDW) state from the Fe sublattice. By chemical doping or applying pressure the magnetism is suppressed and superconductivity can be induced. In most of these FeSCs, superconductivity is found in the proximity of the magnetically ordered state from the Fe sublattice, which leads to the proposal that magnetic fluctuations contribute greatly in the electron paring in the superconducting states (Mazin *et al.*, 2008 ▸; Chubukov *et al.*, 2008 ▸; Kuroki *et al.*, 2008 ▸). Since the discovery of the unconventional FeSCs, Mössbauer spectroscopy has been used widely to study the magnetic state from Fe sites as well as rare-earth elements (Nowik *et al.*, 2011 ▸; Ikeda *et al.*, 2012 ▸). Similarly, SMS experiments probe directly the SDW ordering and can provide insight to the correlation of structural transitions and magnetic transitions under HP–LT conditions (Wu *et al.*, 2013 ▸, 2014 ▸). The strong interplay among magnetism, crystal structure and electronic structure in Fe-based materials can be studied by NRS experiments under HP–LT conditions, with NRIXS providing lattice dynamics at magnetic, structural transitions and superconducting transitions and SMS probing the magnetism.

The partial phonon density of states and magnetic hyperfine fields of Fe were studied in a single-crystalline EuFe_2_As_2_ sample at the resonant energy of 14.41 keV. The ^57^Fe enriched single-crystalline sample was grown at the Institute of Physics, Chinese Academy of Sciences, China. EuFe_2_As_2_ is very unique among the 122 family of Fe-pnictides with regard to its rich magnetic, superconducting and crystal structural transitions when moderate pressure is applied. The pressure–temperature phase diagram of EuFe_2_As_2_ has been studied through electrical resistivity and magnetic susceptibility measurements with oil as pressure medium (Kurita *et al.*, 2011 ▸; Matsubayashi *et al.*, 2011 ▸; Miclea *et al.*, 2009 ▸; Terashima *et al.*, 2009 ▸). At ambient pressure EuFe_2_As_2_ shows antiferromagnetic ordering from the Fe sublattice at 190 K accompanied by a tetragonal to orthorhombic structural transition (Uhoya *et al.*, 2011 ▸), and antiferromagnetic ordering from the Eu sublattice at 19 K. Under pressure the magnetic transitions of the Fe lattice and the structural transition are suppressed. In a narrow pressure range of 2.4–3 GPa the sample was found to be superconducting with *T*_C_ ≃ 30 K (Kurita *et al.*, 2011 ▸; Matsubayashi *et al.*, 2011 ▸; Miclea *et al.*, 2009 ▸; Terashima *et al.*, 2009 ▸). The role of the phonons in the superconducting transition has not been studied.

During our measurements, diamond anvils with 500 µm culet were used to achieve high pressure in the miniature DAC. A Be gasket was pre-indented to 80 µm and a hole of 120 µm diameter was electro-spark drilled through the center to form a sample chamber. Neon was used as pressure medium to achieve a hydrostatic environment around the sample. X-ray beam at 14.41 keV was focused to 15 µm at the sample position. The incoming X-ray beam is along the *c*-axis of the single-crystalline sample. The NRIXS spectra were recorded by two APD detectors shown in Fig. 2 ▸. SMS signals from the sample and the instrumental resolution function were collected by an APD detector in the forward direction. Several ruby spheres were placed next to the sample to allow *in situ* pressure determination at any temperature from the *R*_1_ ruby fluorescence line by an online ruby system using the revised pressure scale by Chijioke *et al.* (2005 ▸).

## 4. Results and discussion   

In the current system, a stabilized base temperature (∼20 K) can be reached within 30 min from room temperature. Two main factors are responsible for this rather high minimal temperature:

(i) As shown in Figs. 2(*a*) and 2(*b*) ▸, the homemade copper DAC holder is screwed onto the cold finger of the cryostat where the liquid helium flow ends. The DAC is clamped onto the holder. The minimal temperature achieved on the DAC is largely affected by the couplings of the holder to the cold finger and the DAC to the holder. The extra junction between the cold finger and the DAC reduces the cooling efficiency shown by the temperature gradient from the cold finger to the DAC: 9 K on the cold finger while 20 K on the diamond anvil cell.

(ii) On the radiation shield two windows were cut to allow the nuclear fluorescence signals to reach the APD detectors through two Kapton windows, each of area 1 cm × 1 cm, on the vacuum shroud. The large windows expose the DAC to thermal radiation from the Kapton window which is at room temperature, greatly increasing the minimal temperature on the DAC.

As a result of using the miniature DAC, a good counting rate from the sample was obtained with a data collection time of 6–8 h at each pressure and temperature. This is comparable with a typical data collection time for a high-pressure NRIXS experiment at ambient temperature. The phonon excitation spectra of a single-crystal Eu^57^Fe_2_As_2_ were measured in the DAC at 0.4 and 3.4 GPa and 24 K. At 0.4 GPa the NRIXS data were collected with an energy range of −20 meV to +80 meV with 0.25 meV step size and 3 s at each energy point. The same energy scan was repeated more than ten times to obtain satisfying statistics on the phonon peaks. The data at negative energies as well as above 50 meV are at the noise level. Therefore the spectrum at 3.4 GPa was collected from −20 meV to 60 mV.

**Figure 3:**
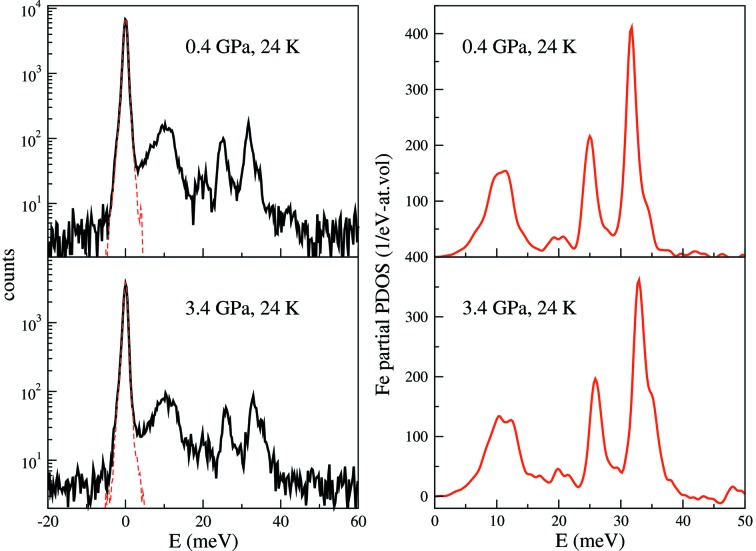
(Left) NRIXS spectra of Eu^57^Fe_2_As_2_ under high pressures and 24 K (solid lines) and instrumental resolution function (red dashed lines) measured by SMS. The intensity of the resolution function is scaled to the elastic peak intensity in the NRIXS spectra. (Right) Derived Fe specific PDOS.

Fig. 3 ▸ shows the NRIXS spectra, the instrumental resolution function and the Fe partial phonon density of states (PDOS) at high pressure and low temperature. The NRIXS spectrum at each pressure is the sum of individual scans. The resolution function is scaled to the elastic peak of the NIRXS spectrum. The measured full width at half-maximum of the resolution function is 1.1 meV. In the NRIXS spectrum the elastic peak is removed based on the instrumental resolution function. The PDOS was extracted from the data using the *PHOENIX* package (Sturhahn, 2000 ▸). The derived Lamb–Mössbauer factors at 0.4 GPa and 3.4 GPa are 0.891 (3) and 0.893 (4), respectively. The PDOS show three major peaks at around 11, 25 and 32 meV, in good agreement with the results of Kobayashi *et al.* (2013 ▸). The peak around 11 meV split into two peaks at 3.4 GPa and 24 K. The peaks around 25 and 32 meV shifted toward higher energy under pressure. The changes of PDOS with pressure and temperature warrant theoretical calculations to understand the origins in terms of magnetic and structural transitions. More detailed discussions of the NRIXS together with SMS results will be published in a separate paper (Bi *et al.*, 2015 ▸).

## 5. Conclusion   

A novel technique of NRIXS under HP–LT conditions has been developed at beamline 3-ID of the APS. The instrument is easy to use with a good counting rate. The new capability of measuring the phonon density of states has been demonstrated with a single-crystalline Eu^57^Fe_2_As_2_ sample. This setup allows us to record NRIXS, SMS and X-ray diffraction data under the same HP–LT conditions simultaneously. Pressure can be determined *in situ* by an online ruby system or X-ray diffraction system. The development significantly broadens the capabilities for NRIXS studies. It enables studies of the phonon contribution to phase transitions, such as magnetic, superconducting and metal–insulator transitions in correlated electron systems under HP–LT conditions. Simultaneous measurements of X-ray diffraction, electrical resistivity and susceptibility should be possible in this setup to gain more accurate information on the *P*–*T* phase diagram.

To further improve the counting statistics, a perforated anvil may be used on the upstream side to reduce the absorption by the diamond anvil at the energy of 14.4 keV which may further reduce the counting time by 40%. This technique can also be used on other resonant isotopes such as ^151^Eu, ^119^Sn and ^161^Dy to study pressure- and temperature-induced transitions. Further development is underway in developing a new cryostat to achieve temperatures as low as 4 K. A gas membrane system will be incorporated to enable *in situ* pressure tuning at low temperature. We realise that this new capability is readily applicable to other X-ray spectroscopy techniques at high pressure and low temperature, such as X-ray emission spectroscopy to determine spin state, X-ray Raman spectroscopy for details of chemical bonding, resonant inelastic X-ray scattering (RIXS) for electronic band structure, and X-ray absorption spectroscopy EXAFS and XANES for atomic near-neighbor environment and valence, respectively.
